# A Review of the Biological Activity of Amidrazone Derivatives

**DOI:** 10.3390/ph15101219

**Published:** 2022-09-30

**Authors:** Renata Paprocka, Małgorzata Wiese-Szadkowska, Tomasz Kosmalski, Daria Frisch, Magdalena Ratajczak, Bożena Modzelewska-Banachiewicz, Renata Studzińska

**Affiliations:** 1 Department of Organic Chemistry, Faculty of Pharmacy, Collegium Medicum in Bydgoszcz, Nicolaus Copernicus University in Toruń, Jurasza Str. 2, 85-089 Bydgoszcz, Poland; 2 Department of Immunology, Faculty of Pharmacy, Collegium Medicum in Bydgoszcz, Nicolaus Copernicus University in Toruń, M. Curie-Skłodowska Str. 9, 85-094 Bydgoszcz, Poland

**Keywords:** amidrazone, aminoguanidine, antibacterial, antifungal, antiparasitic, antitumor, anti-inflammatory

## Abstract

Amidrazones are widely used in chemical synthesis, industry and agriculture. We compiled some of the most important findings on the biological activities of amidrazones described in the years 2010–2022. The data were obtained using the ScienceDirect, Reaxys and Google Scholar search engines with keywords (amidrazone, carbohydrazonamide, carboximidohydrazide, aminoguanidine) and structure strategies. Compounds with significant biological activities were included in the review. The described structures derived from amidrazones include: amidrazone derivatives; aminoguanidine derivatives; complexes obtained using amidrazones as ligands; and some cyclic compounds obtained from amidrazones and/or containing an amidrazone moiety in their structures. This review includes chapters based on compound activities, including: tuberculostatic, antibacterial, antifungal, antiparasitic, antiviral, anti-inflammatory, cytoprotective, and antitumor compounds, as well as furin and acetylocholinesterase inhibitors. Detailed information on the compounds tested in vivo, along the mechanisms of action and toxicity of the selected amidrazone derivatives, are described. We describe examples of compounds that have a chance of becoming drugs due to promising preclinical or clinical research, as well as old drugs with new therapeutic targets (repositioning) which have the potential to be used in the treatment of other diseases. The described examples prove that amidrazone derivatives are a potential source of new therapeutic substances and deserve further research.

## 1. Introduction

**Figure 1:**
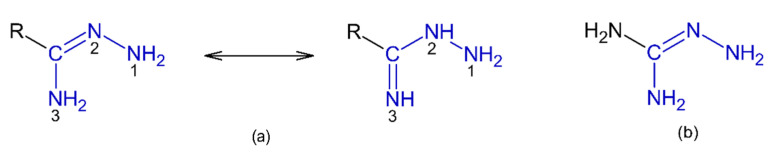
(**a**) The general structure of amidrazones, showing the numbering of the nitrogen atoms and the possible phenomenon of tautomerism. (**b**) The structure of aminoguanidine.

Amidrazones (hydrazones of acid amides) are organic compounds represented by the general structure presented in Figure 1a. These compounds are characterized by three nitrogen atoms (*N^1^*, *N^2^* and *N^3^*), of which only two, *N^1^* and *N^3^*, may be substituted with alkyl or aryl groups. Amidrazones can exhibit tautomerism due to the transfer between the nitrogen atoms *N^3^* and *N^2^* [1,2]. Amidrazones are monoacid bases which form salts with inorganic acids, among which the most widely known are the hydrochlorides [2].

Amidrazones constitute a group of interesting compounds used mainly as precursors for the synthesis of five-, six- and seven-membered heterocyclic systems. Simple methods of obtaining 1,2,4-triazole, thiatriazole and 1,2,4-triazine derivatives [3], tetrazole [4] derivatives and other derivatives [1] from amidrazones have been described previously. Due to the presence of nitrogen atoms, amidrazones can form complexes with transition metals [2].

The nomenclature of amidrazones has evolved in recent years. In older papers, amidrazones are named after the acid theoretically obtained from them by hydrolysis (e.g., CH_3_C(=NNH_2_)NH_2_ is acetamidrazone) [1,2]. Currently, the International Union of Pure and Applied Chemistry (IUPAC) recommends a different numbering and nomenclature of amidrazones (R-C(=N-NH_2_)-NH_2_ as carbohydrazonamides and R-C(=NH)-NH-NH_2_ as carboximidohydrazides [5]). However, the previous nomenclature is still widespread in many published papers. For example, on sciencedirect.com, in 2010–2021, the word “amidrazone” gave 243 results, while “carbohydrazonamide” gave only 15. Therefore, in this work, the original nomenclature and numbering of the nitrogen atoms in amidrazones were adopted (Figure 1a).

Aminoguanidine (NH_2_)_2_-C=N-NH_2_ (Figure 1b) is a simple, non-toxic compound that is closely related to amidrazones. Some authors of older publications do not classify it among the amidrazones [2], while others do consider it an amidrazone [1]. Taking into account the similarity of aminoguanidine to amidrazones in terms of its structure, application in the synthesis of heterocyclic compounds and the biological activities of the obtained products, in this work, aminoguanidine and its derivatives are presented among the amidrazone derivatives. 

Many amidrazones and their derivatives exhibit a broad spectrum of biological activities, e.g., antibacterial [6], antifungal [6,7], antimalarial [8], antiviral [9], anti-inflammatory [10], analgesic [10], anticonvulsant [11] and insulin-mimetic [12], and as thrombin inhibitors [13]. Despite the presence of some review articles on amidrazone chemistry [1,2], a comprehensive study of the biological activity of amidrazones is still lacking. The last review concerning the biological activities of aminoguanidine derivatives was published back in 2009 [14], which justifies the presentation of the up-to-date information in this field. In addition, the diversity of the nomenclature used in medicinal chemistry literature for amidrazone derivatives (i.e., amidrazones, carbohydrazonamides, aminoguanidines, guanidines, amidinohydrazones, hydrazones, hydrazidines and others) makes it difficult for researchers to discover information about the biological activities of these compounds by using keywords, in the case of a person who is unfamiliar with the subject. Therefore, a double search strategy was used in the search for articles, using both keywords (amidrazone, carbohydrazonamide, carboximidohydrazide, aminoguanidine) and structure strategies. The best selected compounds with significant biological activities were included in the review. The data were obtained using the ScienceDirect, Reaxys and Google Scholar searching engines. 

This work encapsulates some of the most important findings on the biological activities exhibited by amidrazone derivatives described from 2010–2022. The described structures derived from amidrazones include: (a) amidrazone derivatives; (b) aminoguanidine derivatives; (c) complexes obtained using amidrazones as ligands; and (d) some examples of cyclic compounds obtained from amidrazones and/or containing an amidrazone moiety in their structures (e.g., **1**, **32**, **51**). We also discuss their toxicity, mechanism of action and potential use in preclinical trials. 

## 2. Results

### 2.1. Antimicrobial Activity

#### 2.1.1. Tuberculostatic Activity

**Figure 2:**
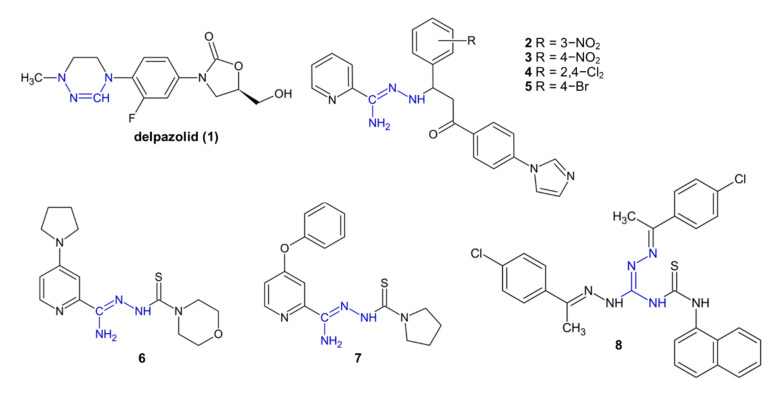
The structures of tuberculostatic compounds **1**–**8**.

Delpazolid (**1**, Figure 2), also called LCB01 0371, was the first compound containing a cyclic amidrazone moiety that was developed to treat multi-drug-resistant tuberculosis. Delpazolid successfully passed the phase I clinical trials, confirming its safety (maximum tolerated dose in humans = 2400 mg) [15]. A phase II study is currently recruiting, which explores the combination of delpazolid with bedaquiline, moxifloxacin and delamanid in patients with newly diagnosed, uncomplicated, drug-sensitive pulmonary tuberculosis [16].

Compounds **2**–**5**, which possess a 2-pyridylamidrazone moiety, demonstrated tuberculostatic activity against *Mycobacterium gordonae* (MIC = 2–8.8 µM). Derivatives **2**–**3** inhibited the growth of *M. tuberculosis* (MIC = 4.4 µM). Interestingly, compounds **4**–**5**, substituted with chloride or bromide atoms instead of nitro group, were even 7-fold more active against *Mycobacterium kansasii* than isoniazid (MIC = 4.2 µM) [17].

Another 2-pyridylamidrazone derivatives, **6** and **7**, showed a strong tuberculostatic activity against the standard H37Rv strain and clinically isolated drug-resistant *M. tuberculosis* strains (MIC = 0.4 µg/mL) [18].

Derivative **8**, containing an aminoguanidine moiety, showed strong tuberculostatic activity against (MIC = 0.78 µM), and low cytotoxicity to, human embryonic kidney cells. The mechanism of **8** was the inhibition of the enoyl acyl carrier protein reductase enzyme (InhA), which was confirmed in vitro and in computational studies [19]. 

#### 2.1.2. Antibacterial Activity

**Figure 3:**
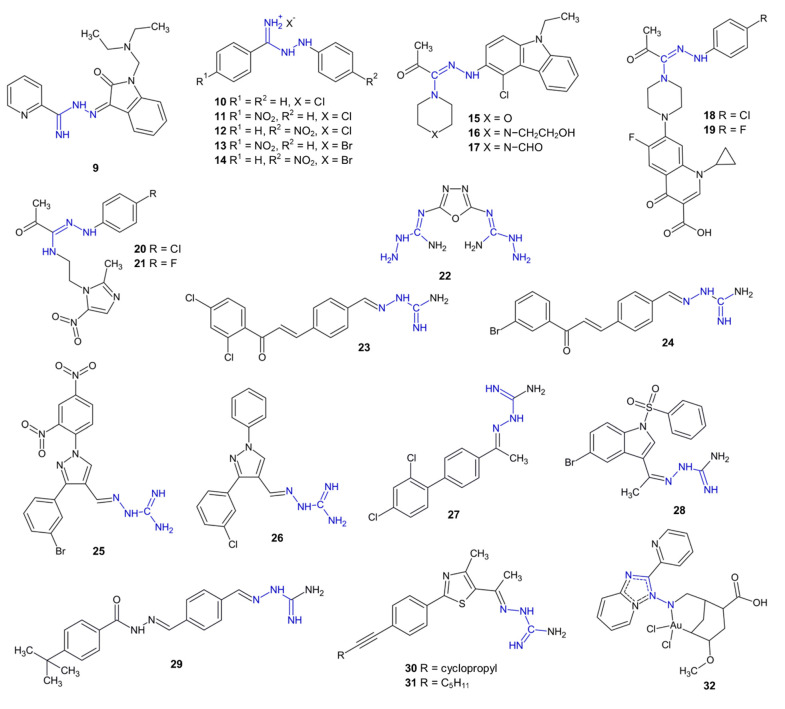
The structures of antibacterial compounds **9**–**32**.

Several compounds with antibacterial activities are presented in Figure 3. 

The previously mentioned compounds **6**–**7** exhibited a significant antibacterial activity against several Gram-positive bacterial strains (*Staphylococcus epidermidis, Micrococcus luteus, Bacillus subtilis, Bacillus cereus* and *Streptococcus mutans*), with MIC values of 0.12–1.95 µg/mL. Additionally, derivative **6** showed an activity against *Staphylococcus aureus* comparable to ciprofloxacin and vancomycin. Interestingly, the replacement of the pyrrolidine ring found in compound **6** with a morpholine moiety present in compound **7** resulted in an approximately twofold decrease in its anti-tuberculosis and antibacterial activities against Gram-positive strains in comparison with the starting compounds of **6**–**7** [18]. Compound **9**, containing an isatin moiety, demonstrated stronger antibacterial activity against *S. aureus* (MIC = 4 µg/mL) than ciprofloxacin [20]. 

The chloride or bromide salts of (*N*^1^-phenyl)phenylamidrazone (**10**) and its derivatives, **11**–**14**, showed antimicrobial activity. The strongest bactericidal activity against *S. aureus* was demonstrated by compounds **12** (minimal bactericidal concentration MBC = 4 µg/mL) and **14** (MBC = 8 µg/mL), while derivatives **10**, **11** and **13** showed similar activity to nifuroxazide (MBC = 16 µg/mL) [21]. 

Among the *N*^1^-(carbazol-3-yl) substituted amidrazones **15**–**17**, compound **15**, with incorporated morpholine, was bacteriostatic (MIC = 1.56 µg/mL) against *B. cereus* [22]. Compound **16** showed bactericidal activity against standard *S. aureus* and clinically isolated MRSA strains (MBC = 3.125 µg/mL). Compound **17** exhibited antibacterial activity against the Gram-negative strain of *Klebsiella pneumoniae* (MBC = 6.25 µg/mL and MIC = 3.125 µg/mL) [22].

Another method of amidrazone modification is the creation of their hybrids with antimicrobial drugs, i.e., ciprofloxacin (**18**–**19**) or metronidazole (**20**–**21**). Compounds **18**–**19** showed antibacterial activity against *Escherichia coli* (MIC_50_ = 0.2 µg/mL), *Pseudomonas aeruginosa* (MIC_50_ = 6.25 µg/mL), *Helicobacter pylori* (MIC_50_ = 4 µg/mL) and *S. aureus* (only **18**, MIC_50_ = 6.25 µg/mL). However, both compounds were less active than ciprofloxacin alone [23]. Amidrazones **20** and **21** showed selective activity against metronidazole-resistant *H. pylori* (MIC = 8 and 16 µg/mL, respectively) [23]. 

Among the aminoguanidine derivatives **22**–**31**, the 1,3,4-oxadiazole derivative **22** showed strong antibacterial activity against Gram-negative *E. coli* and *Salmonella typhimurium* and the Gram-positive *S. aureus, Enterococcus faecium* and *Streptococcus agalactiae* bacterial strains [24].

The chalcone-incorporated derivatives **23**–**24** showed a wide range of antimicrobial activities against *S. aureus*, *S. mutans*, MRSA*, E. coli*, *S. typhimurium* and *P. aeruginosa* (MIC = 1–8 µg/mL) [25].

The 1,2-diazole derivatives **25**–**26** showed strong antimicrobial activity (MIC = 1–4 µg/mL) against Gram-positive (*S. aureus*, MRSA, quinolone-resistant *S. aureus*, *S. mutans*) and Gram-negative (*E. coli*, *S. typhimurium*) bacterial strains [26].

Aminoguanidine derivative **27** demonstrated a wide range of antimicrobial activities, with an MIC value of 1 µM/mL against eight strains (including *S. aureus*, *S. mutans*, *E.coli*, *C. albicans*, MRSA and *Quinolone-resistant S. aureus)*. The inhibition of the dihydrofolate reductase (DHFR) protein is a possible mechanism of action of **27** [27].

Aminoguanidine derivative **28** showed stronger antibacterial activity towards multidrug-resistant strains (*S. aureus*, *E. coli*, MIC = 0.56–2.24 µmol/L) than the five antibiotics used (gatifloxacin, moxiflocaxin, norfloxacin, oxacillin, and penicillin), as well as low cytotoxicity to normal HEK 293T cells. The activity of **28** could be connected to its binding to the *E. coli* FabH-CoA receptor [28].

Aminoguanidine derivative **29** showed antibacterial activity against *B. subtilis* (MIC = 4 µg/mL) and eight other bacterial strains (MIC = 4 µg/mL). The mechanism of action of **29** was its interaction with β-ketoacyl-acyl carrier protein synthase III (FabH) [29].

Thiazole derivatives **30**–**31** demonstrated strong bactericidal activity against the *S. aureus*, MRSA and VRSA bacterial strains (in most cases, MIC = MBC = 2 µg/mL) and were active against MRSA in several animal models. Compound **30** demonstrated resistance to the microsomal cytochrome P450 and stability during metabolism. However, it interacted with enzymes connected to bacterial wall synthesis (such as undecaprenyl diphosphate synthase and undecaprenyl diphosphate phosphatase). Due to its similar activity (but in lower doses) to that of vancomycin in mice, compound **30** may be a new leading structure in the treatment of drug-resistant bacterial strains [30].

Gold(III) complex **32** obtained by the reaction of amidrazone with HAuCl_4_, showed antibacterial activity against *S. aureus* (MIC = 4 µg/mL) and lower toxicity to mice fibroblasts (IC_50_ = 41.8 µg/mL), which suggests the good selectivity of this compound [31].

#### 2.1.3. Antifungal Activity

Among the previously mentioned amidrazone derivatives **10**–**14**, the strongest fungistatic activity against *C. albicans* was exhibited by compounds **11** (MIC = 4 µg/mL) and **10** (MIC = 8 µg/mL). Additionally, derivative **11** was fungicidal at a concentration of 16 µg/mL against *Aspergillus niger* and *Aspergillus brasiliensis* [32]. The presence of a nitro group in the position R^1^ of compound **11** seems to increase its antifungal activity. Contrarily, the addition of a four-nitro substituent in the *N*^1^-phenyl rings of compounds **12** and **14** decreased their antifungal properties but elevated their antibacterial activity. 

The also previously mentioned aminoguanidine derivatives **23**–**27** showed strong antifungal activity against *C. albicans* (MIC = 1–8 µg/mL) [25,26]. The strongest effect on this fungal strain was observed for derivative **22**, containing two aminoguanidine groups (MIC = 0.015–0.5 µg/mL, MBC = 0.031–1 µg/mL) [24].

**Figure 4:**
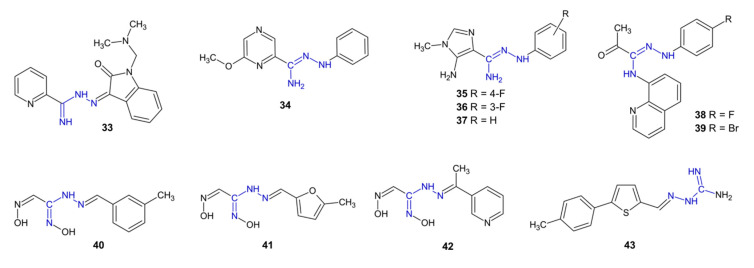
The structures of antifungal compounds **33**–**43**.

Compound **33** (Figure 4) exhibited antifungal activity against *Candida albicans* (MIC = 16 µg/mL) [26]. Pyrazinylamidrazone **34** exhibited antifungal activity against the clinical strain *C. albicans* (MIC = 16 µg/mL). The replacement of the phenyl ring of compound **34** with a hydrogen or a methyl group resulted in the total disappearance of the antifungal activity of the obtained derivatives, which underlines the importance of the phenyl substituent in this position [33].

The imidazolylamidrazone derivatives **35**–**37** demonstrated fungistatic activity against *Candida krusei* (MIC = 3.1–6.3 µg/mL) and *Candida neoformans* (MIC = 2–4 µg/mL) [34]. Derivatives **35**–**37** also displayed a strong inhibitory effect on biofilm development in the case of *Candida* spp. biofilms on nanohydroxyapatite substrate, and the strongest effect was observed for compound **36** [35]. The mechanism of action of compounds **35**–**37** seems to be connected with the production of reactive oxygen species [36]. Amidrazone-quinolone hybrids **38**–**39** showed an antifungal activity in vitro against *C. albicans* comparable to that of fluconazole [37].

Among compounds **40**–**42** (which can also be classified as vic-dioximes), derivative **40** showed a stronger activity than nystatin against the *C. glabrata*, *C. utilis* and *S. cerevisiae* fungal strains (in all cases, MIC = 4 µg/mL) [38]. Compound **41**, which contains a methylfuryl moiety instead of a methylphenyl moiety, demonstrated less antifungal activity against *S. cerevisiae* (MIC = 16 µg/mL) than **40**, along with antibacterial activity against *B. cereus* (MIC = 8 µg/mL) and *Streptococcus pneumoniae* (MIC = 16 µg/mL). Derivative **42**, which possesses a pyridine ring, was selective to the *Candida tropicalis* fungal strain (MIC = 8 µg/mL) [39]. 

Compound **43** showed antifungal activity against *C. albicans, C. krusei, Microsporum canis* and *Trichophyton mentagrophytes* (MIC = 0.5–3.9 µg/mL) and a lower toxicity to danio zebrafish than voriconazole [40].

### 2.2. Antiparasitic Activity

**Figure 5:**
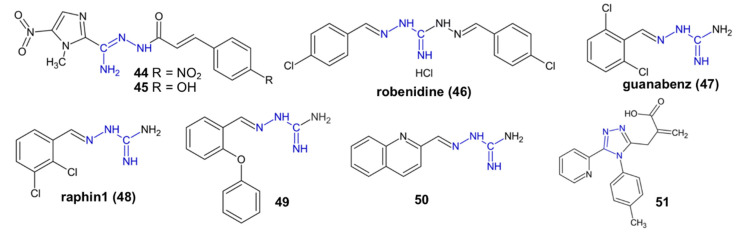
The structures of antiparasitic compounds **44–51**.

In an attempt to obtain antiparasitic agents, amidrazones were enriched with benznidazole (**44**–**45**), metronidazole (**20**–**21**) or ciprofloxacin (**18**) moieties. Derivatives **44** and **45** (Figure 5) demonstrated similar activities to benznidazole against the trypomastigota forms of *Trypanosoma cruzi* (IC_50_ = 9.5 and 12.85 µM, respectively; benznidazol IC_50_ = 10.26 µM). Both compounds were selective to parasite cells, especially derivative **45**, with a selectivity index value of about 33 [41]. Compounds **18** and **21** were revealed to possess an antitrichomonal activity about two times stronger than that of metronidazole against *Trichomonas vaginalis* [34]. Compound **20** showed antigiardial activity comparable to metronidazole against *Giardia lamblia* (IC_50_ = 5.6–7.2 µg/mL) [23].

Likewise, aminoguanidine derivatives **46**–**50** were studied as antiparasitic agents. Robenidine (**46**) is an antibiotic used in veterinary medicine which, in current research, has shown an antigiardial activity against *G. lamblia* comparable to that of metronidazole. In contrast to the reference drug, compound **46** completely inhibited the adherence of trophozoides and is a candidate for a new generation of antigiardial drugs [42]. 

Guanabenz (**47**) is a known antihypertensive drug currently drawing attention for the purpose of other medicinal uses. It has exhibited antiparasitic activity against the replicative stages of *Toxoplasma* and *Plasmodium falciparum* [43]. Guanabenz inhibited the *Toxoplasma* dephosphorylation enzyme eIF2α. This translational control is critical during infections with both the replicative and latent forms of *Toxoplasma* [43,44]. In mice models, guanabenz extended the survival of mice acutely infected with *Toxoplasma* within 2–3 days [44] and reduced the number of brain cysts in chronically infected mice [43].

Aminoguanidine derivatives **48**–**50** showed antileishmanial activity against amastigotes of *Leishmania chagasi* (IC_50_ = 0.6–7.27 μM) comparable to pentamidine (IC_50_ = 4.4 μM). Compounds **48**–**50** showed a 50–80 times higher toxicity to amastigotes than to murine macrophages. The mechanism of action of the most promising compound, **50**, is probably related to its interaction with the active site of the trypanothione reductase enzyme, interfering in the redox system of *L. chagasi* amastigotes [45].

The 1,2,4-triazole derivative **51**, obtained from amidrazone, showed strong anthelmintic activity (2.475 µg/µL) against *Rhabditis* nematodes. Due to its stronger activity than albendazole and low toxicity to PBMC, compound **51** could be a candidate for the development of new anthelmintic drugs [46]. 

### 2.3. Antiviral Activity

**Figure 6:**
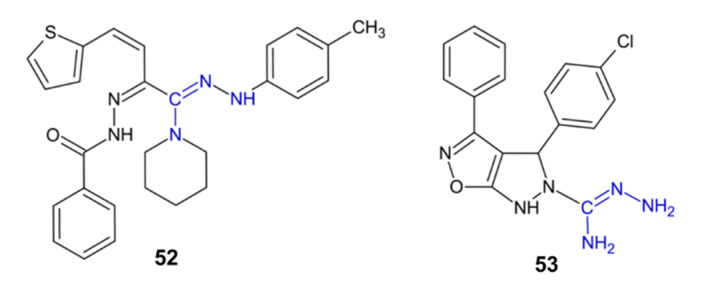
The structures of antiviral compounds **52**–**53**.

Amidrazone derivative **52** (Figure 6) reduced the number of plaques of herpes simplex type-1 (HSV-1) on Vero cells by 67% [47]. Amidrazon **53**, with a pyrazoloisoxazole moiety, showed antiviral activity against two HIV strains studied in two leukemia cell lines (EC_50_ = 0.17–0.46 nM). Compound **53** was two times more effective than the anti-HIV drug efavirenz and about two times less toxic to uninfected cell lines. Compound **53** exhibited strong inhibitory activity towards HIV reverse transcriptase (HIV-RT). Molecular docking confirmed that compound **53** strongly interacts with the HIV-RT active pocket, which enables its classification as a potential non-nucleoside reverse transcriptase inhibitor [48]. 

### 2.4. Anti-Inflammatory Activity

**Figure 7:**
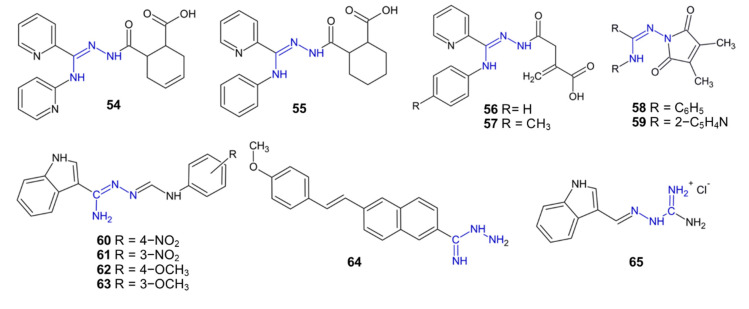
The structures of anti-inflammatory compounds **54**–**65**.

Derivatives of *N*^1^,*N^3^*-substituted 2-pyridylamidrazone **54**–**57** (Figure 7) were studied in order to assess their anti-inflammatory activity in mitogen-stimulated peripheral blood mononuclear cells (PBMC). Compound **54** decreased the production of TNF-α by 43% and showed no toxicity to PBMC at a concentration of 100 µg/mL [49].

Compound **55**, at a concentration of 10 µg/mL, inhibited the production of the pro-inflammatory cytokine IL-6 by 35% [50]. The median lethal dose of **55** (*i.p.*) in mice was identified as 417 mg/kg. Compound **55**, at a concentration of 21 mg/kg, reduced rat hind paw edema to a greater extent than diclofenac at a dose of 50 mg/kg. Moreover, derivative **55** demonstrated antinociceptive activity in mice comparable to that of morphine but with a longer duration of action. In summary, compound **55** could be a potential non-steroidal anti-inflammatory drug [50].

Compound **56**, at a concentration of 10 µg/mL, inhibited the production of TNF-α in PBMC stimulated by lipopolysaccharide (LPS) by 53% [51]. Compound **57**, at a concentration of 50 µg/mL, showed no toxicity but strongly inhibited the proliferation of PBMC activated by anti-CD3 antibodies or phytohaemagglutinin by 90–99%, and the observed effects were comparable to or stronger than those of ibuprofen. The mechanism of action of derivative **57** is cell cycle arrest at the G1 phase [52].

Additionally, some 1,2,4-triazole derivatives obtained by the cyclisation of amidrazones, similar to **56**–**57**, showed a strong significant anti-inflammatory activity comparable to ibuprofen’s inhibition of PHA-stimulated PBMC proliferation and TNF-α production [46,53].

Anti-inflammatory activity was also reported for amidrazone-derived pyrrole-2,5-dione derivatives **58**–**59**. Compound **58**, possessing two phenyl substituents, significantly reduced the production of IL-6 (by 64%) in LPS-stimulated PBMC cultures. Both compounds **58** and **59** inhibited the proliferation of PBMC even at a low dose of 10 µg/mL, and the strongest effect was observed for the latter, possessing two 2-pyridine rings [54].

The previously mentioned *N*^1^,*N^3^*-substituted amidrazones **38**–**39** showed an anti-inflammatory activity in protein denaturation assays comparable to that of the sodium salt of diclofenac. Both derivatives showed a stronger antioxidant activity than ascorbic acid [37]. 

Indoleamidrazone derivatives **60**–**63** produced a stronger reduction in carrageenan-induced rat paw edema in rats than indomethacin. In general, compounds possessing nitro or methoxy substituents at the para position showed stronger anti-inflammatory effects than derivatives possessing the same groups in the meta position [55]. 

Naphthylamidrazone derivative **64** revealed properties preventing the adverse effects of a chronic inflammatory reaction in the articular chondrocytes through a mechanism involving the ASIC1a channels, which are sensitive to the acidification of the environment. Compound **64**, in a concentration range of 6.25–50 µM, caused a significant inhibition of the ASIC1a protein expression in the joint chondrocytes comparable to amiloride (a weak non-selective ASIC1 inhibitor). Additionally, compound **64,** at a dose of 25 μM, decreased the number of Ca^2+^ ions in the acidic environment of isolated rat articular chondrocytes by 69%, which is almost three times higher than the effect of amiloride at a dose of 100 μM. In summary, it can be stated that compound **64** is a potential drug for rheumatoid arthritis [56]. 

Aminoguanidine (**AG**) has been shown to possess strong anti-inflammatory and antioxidant activities in multiple ways. It inhibits the formation of highly reactive advanced glycosylation end products in the course of advanced diabetes. **AG** passed phase III clinical trials in diabetic patients. Although high doses of **AG** induced side effects, including liver dysfunction, low doses of **AG** therapy could be promising for the treatment of renal diseases [57].

Aminoguanidine derivatives **23**–**26** were studied in tests on xylene-induced ear edema in mice. Compound **23** showed an anti-inflammatory activity similar to indomethacin. However, compound **24**, with a bromine atom at position 3, was about two times less effective [25]. Derivatives **25** and **26** were about two times stronger as anti-inflammatory agents than indomethacin [26].

Aminoguanidine derivative **65** was studied in an LPS-stimulated neonatal sepsis mice model. The mechanism of compound **65** was connected to a decreased pro-inflammatory cytokine release and COX-2 expression, as well as the suppression of microglia activation. Additionally, septic mice treated with derivative **65** did not exhibit the cognitive impairment and the anxiety behavior caused by LPS [58].

### 2.5. Cytoprotective Activity

**Figure 8:**
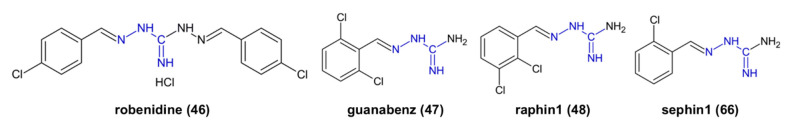
The structures of cytoprotective compounds **46**–**48** and **66**.

Some aminoguanidine derivatives, such as guanabenz (**47**), sephin1 (**66**) and raphin1 (**50**), possess cytoprotective activities (Figure 8). The effects of those compounds are connected with the reduced deposition of proteins of abnormal conformation, which are present in many neurodegenerative diseases, such as Alzheimer’s, Parkinson’s, amyotrophic lateral sclerosis (ALS) and others. Guanabenz and sephin1 are inhibitors of the stress-induced transcription factor R15A. They prolong eIF2α (translation initiation factor) phosphorylation and, in consequence, cause the transient attenuation of protein synthesis induced by endoplasmic reticulum (ER) stress [59]. Guanabenz is currently in clinical trials as a method for the management of multiple sclerosis [60] and amyotrophic lateral sclerosis [61,62]. Guanabenz has also been shown to reduce neuroinflammation in mice with latent toxoplasmosis and reversed the behavioral changes in the studied rodents [63]. Sephin1 has passed phase I clinical trials and is being developed for treating Charcot-Marie-Tooth disease [64]. Moreover, sephin1 showed protective activity in a mouse model of multiple sclerosis [65].

Raphin1 is an inhibitor of the constitutively expressed transcription factor R15B, which may be useful when combating a wide range diseases, as it could enable the increase in the control capacity of the protein quality by transiently increasing eIF2α phosphorylation and translation attenuation. It was effective in a mouse model of Huntington’s disease [66]. Moreover, the previously mentioned robenidine showed cytoprotective properties [67]. 

### 2.6. Antitumor Activity

**Figure 9:**
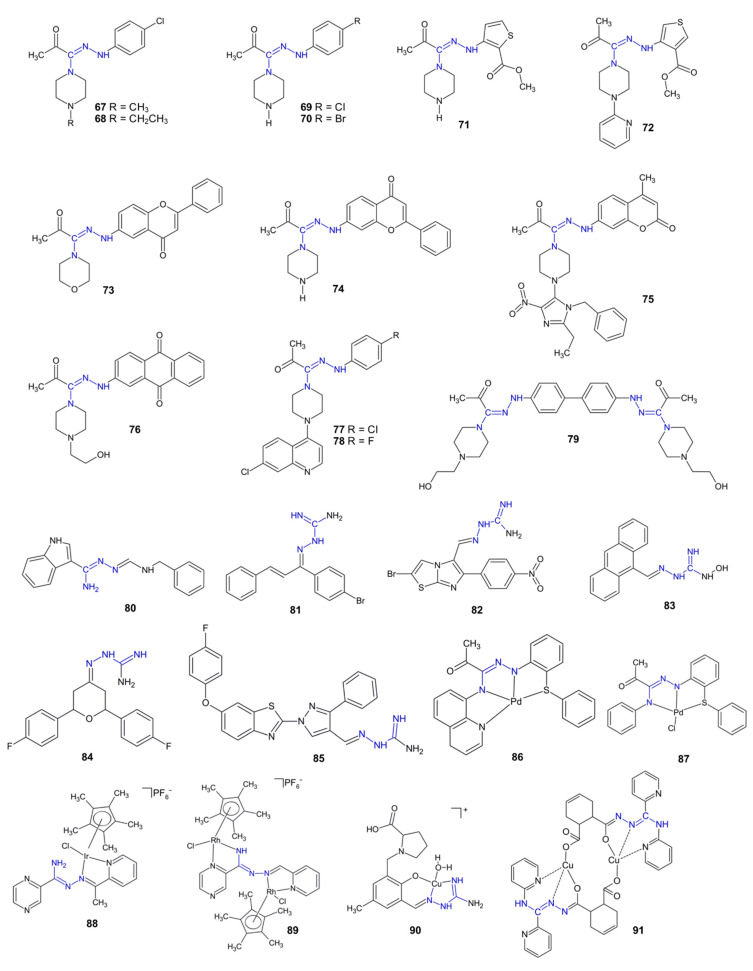
The structures of antitumor compounds **67**–**91**.

Many piperazine-incorporating amidrazones, including **18**–**19**, **67**–**72** and **74**–**79** (Figure 9), were studied as antineoplastic agents. Compounds **67** and **68**, in a panel of 55 different cancer cell lines, produced medium IC_50_ values of 4.81 µM and 4.92 µM, respectively, which were similar to the values of the total growth inhibition (TGI = 4.47 and 4.52 µM, respectively). This underlines their strong anti-cancer properties [68]. Moreover, amidrazones **69**–**70** showed antiproliferative activity against several cancer cell lines, including leukemia K562, breast MCF-7 (Table 1), prostate PC-3 and colon HCT (in all cases, IC_50_ = 1.9–3.9 µM) [69]. 

Amidrazones possessing a thiophenyl (**71**–**72**), flavone (**73**–**74**) or coumarin (**75**) moiety, as well as bisamidrazone derivative **79**, showed antiproliferative activity against the MCF-7 and K562 cancerous cell lines (Table 1). Compounds **72**, **76** and **79** had low toxicity to human fibroblasts in vitro. Molecular docking revealed a similarity of compounds **72**–**76** with imatinib (a drug belonging to the group of tyrosine kinase inhibitors) during interactions with bcr-abl tyrosine kinase, which may indicate a similar mechanism of action of those compounds [70,71,72,73,74,75,76]. Alternatively, according to in silico studies, derivative **79** could act as an effective inhibitor of phosphatidylinositol 3-kinase, the hyperactivity of which was observed in cells of the MCF-7 line [77]. 

**Table 1:** IC_50_ values of select compounds against MCF-7 and K562 cancerous cell lines.

Comp.	IC_50_ MCF-7	IC_50_ K562	Ref.
**69**	2.50 µM	3.10 µM	[69]
**70**	2.70 µM	3.50 µM	[69]
**71**	7.26 µM	9.91 µM	[70]
**72**	>50 µM	1.02 µM	[71]
**73**	5.18 µM	2.89 µM	[72]
**74**	5.91 µM	5.02 µM	[73]
**75**	20.20 µM	9.30 µM	[74]
**76**	4.50 µM	1.10 µM	[75]
**79**	4.30 µM	3.00 µM	[77]
**81**	0.09 µM	-	[78]

Ciprofloxacin derivatives **18**–**19** showed antiproliferative activity against the HeLa and MCF-7 cancerous cells [23]. Amidrazones **78**–**79**, which possess a chloroquine moiety, showed antiproliferative activity against the cervix HeLa and MCF-7 cancer cells [23]. 

Indoleamidrazone **80** inhibited the proliferation of MCF-7 cells by 68% at a concentration of 100 µg/mL [55]. As previously mentioned, the similar compounds **60**–**63,** which possess nitro- or methoxy-phenyl substituents instead of the benzyl observed in **80**, were inactive, except for derivative **63**, which showed a 61.5% growth inhibition of MCF-7 cells [55]. 

Aminoguanidine derivative **81** demonstrated strong antiproliferative activity against MCF-7 and an inhibitory effect on tubulin polymerization (IC_50_ = 8.4 µM). Molecular docking revealed that the probable mechanism of derivative **81** may be connected with colchicine biding [78]. Compound **82** showed a potent inhibition of ribosomal kinase RSK2 and MCF-7 tumor cell growth inhibition [79].

Computational methods were used to identify compounds with anticancer properties. Aminoguanidine derivative **83** was one of the predicted compounds, with a confirmed antiproliferative activity against HL-60 leukemia cells (IC_50_ = 11 µM) and low to towards Vero cells (IC_50_ > 100 µM) [80].

Compound **84** showed antiproliferative activity against the HL-60, K562 and HT-29 cell lines (IC_50_ = 8.9–12.5 µmol/L), and it was more effective than etoposide against the latter two lines [81]. Compound **85** showed high antitumor activity against the MDA-MB-231, MCF-7, HEP-G2 and SMMC-7721 cancer lines (IC_50_ = 2.31–3.75 µM). Compound **85** induced apoptosis by downregulating Bcl-2 and upregulating Bax protein levels in MDA-MB-231 cancer cells [82]. 

Pd(II) complex **86** showed high cytotoxicity to various cancerous cell lines, including DU-145, MCF-7, HCT-116 and breast MDA231 (IC_50_ = 0.143–0.492 µM). However it was not toxic to skin fibroblasts [83]. The similar Pd(II) complex **87** showed also antiproliferative activity towards MCF-7 and T47D breast cancer lines and very low cytotoxicity to normal Vero cells [84]. Complexes **88**–**89** showed cytotoxic activity against HT-29, HCT-116 ^+/+^ and HCT-116^−/−^, as well as selectivity to cancerous cells [85]. 

Cu(II) complex **90** showed antiproliferative activity against the Colo-205 adenocarcinoma cell line and low toxicity to MRC-5 human lung fibroblasts [86]. Another Cu(II) complex, **91**, at concentration 100 µg/mL, showed a similar (almost total) antiproliferative activity to cisplatin against colon CX-1 and colon SW-948 cancer and epidermal A431 cell lines but was about 12-fold less toxic than the reference drug [49].

In 2022, two publications describing the antitumor activity of *N*^1^-benzylidenepyrazine-2-carbohydrazonamide complexes were published. The strongest activity was reported for the cobalt complex against glioma U87 MG cancerous cells (IC_50_ = 7.69 µg/mL) [87,88]. However, the structures of those complexes have not been precisely specified.

### 2.7. Furin Inhibition

**Figure 10:**
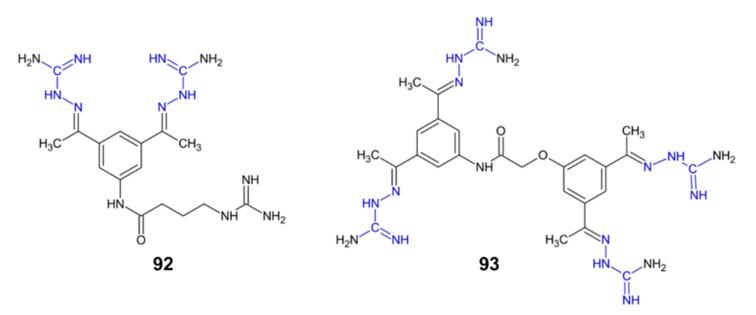
The structures of furin inhibitors **92**–**93**.

Furin is a trans-membrane protein which plays an important role in many bacterial and viral diseases, tumorigenesis, neurodegenerative disorders and diabetes [89]. It has recently been shown that furin inhibitors can be used to successfully block the entry of the SARS-COV-2 virus [90]. Aminoguanidine derivatives **92** and **93** (Figure 10) showed furin inhibitory activity (K*_i_*= 0.46 µM and 0.58 µM, respectively). Additionally, derivative **92** also showed inhibitory activity against trypsin, while compound **93** was also a thrombin inhibitor [89].

### 2.8. Acetylocholinesterase Inhibition

**Figure 11:**
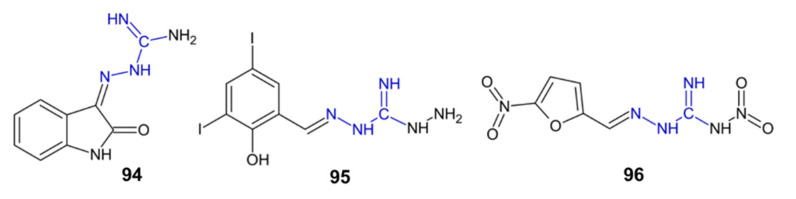
The structure of compound **94**–**96**.

Several compounds were identified as potential acetylcholinesterase (AChE) or butyrylocholinesterase (BChE) inhibitors in the search for potential drug candidates for treating Alzheimer’s disease (Figure 11, Table 2). Compound **75** showed high activity against, and selectivity to, BChE and was about 3900 times stronger in its activity against this enzyme than tacrine [91]. 

Aminoguanidine derivative **94** showed a threefold stronger AChE inhibitory activity than rivastigmine and no selectivity towards BChE. Compound **95** was a selective inhibitor of BChE, with an approximately 16-fold lower AChE inhibitory activity, while derivative **96** was a selective AChE inhibitor. This proves the great potential of aminoguanidine derivatives, which may, in the future, act as inhibitors of various types of cholinesterases [92].

## 3. Summary

We compiled the biological activities of amidrazone derivatives described in the years 2010–2022. Antimicrobial, antitumor, anti-inflammatory and antiparasitic activities constitute the main kinds of exhibited biological activities. The most important compounds studied in vitro are presented in Table 3, together with their activity details. Due to their advanced stages in preclinical studies, they form an important group, from which new therapeutic substances may emerge. Compounds with known mechanisms of action are summarized in Table 4. 

Among the antimicrobial agents, delpazolid showed a low toxicity and high efficacy and is undergoing further clinical trials for the treatment of tuberculosis. The 2-pyridylamidrazone moiety determines the anti-mycobacterial properties of compounds **2**–**7**. It is worth noting that the amidrazones with the unsubstituted nitrogen *N*^3^ (**2**–**7**, **9**–**14** and **33**–**37**) showed stronger antimicrobial properties than amidrazones **54–55**, which are *N*^3^-substituted with aryl rings [49,50]. In general, aminoguanidine derivatives **22**–**31** revealed a wider range of antimicrobial activities, as well as stronger antibacterial and antifungal properties than amidrazones **9**–**21**. Moreover, derivative **22**, which possesses two aminoguanidine groups, showed the strongest antimicrobial effects. Aminoguanidine derivatives **30**–**31** showed significant antibacterial effects in various animal models and deserve further research. 

Eight derivatives (**23**, **25**–**26**, **55**, **60**–**63**) showed significant anti-inflammatory activity in rodents. Moreover, the anti-inflammatory effect of compound **65**, used in the research on the treatment of neonatal anti-sepsis in mice, deserves greater attention.

Amidrazones demonstrated a diverse number of antitumor mechanisms, acting as brc-abl kinase inhibitors (**72**–**76**), an inhibitor of phosphatidylinositol 3-kinase (**79**), an inhibitor of tubulin polymerization (**81**) and an inhibitor of ribosomal kinase RSK2 (**82**), which indicates their potential in the search for new anti-cancer drugs. Compound **72** showed the highest selectivity and may be a future drug candidate for leukemia. 

Aminoguanidine derivatives exhibited cytoprotective activity and inhibited cholinesterases. Their possession of both these mechanism simultaneously could be useful in the search for a cure for Alzheimer’s disease. The phosphorylation of eIF2α translation initiation factor by guanabenz, sephin1 or raphin1 is promising in regard to the prevention and treatment of many neurodegenerative diseases. For example, guanabenz, an old-generation antihypertensive drug, is currently being studied for new potential medical applications, including the treatment of amyotrophic lateral sclerosis, multiple sclerosis and parasitic toxoplasmosis. 

Amidrazones showed moderate toxicity in various models (Table 5). However, among the derivatives with the lowest toxicity, as many as five (**44**–**45, 56**–**57** and **59**) contain an acyl group at atom *N*^1^, which may be valuable for the synthesis of new derivatives with more advantageous properties. 

A useful property of amidrazones is their use as ligands for the synthesis of complexes with metals, which provides researchers with the opportunity to obtain new compounds with anti-tumor (e.g., **86**) or antibacterial (**32**) properties.

## 4. Conclusions

Amidrazones remain an interesting area for researchers, as evidenced by the latest works from 2022. Many derivatives described in this review show strong biological activities and deserve more detailed research in this field. We hope that this article, which systematizes the knowledge about the biological activities of amidrazones, will increase the scientific interest in these compounds and, in effect, will encourage the development of novel derivatives and their introduction to research in preclinical and clinical studies.

## Figures and Tables

**Table 2:** Inhibitory activity of select compounds against AChE and BChE.

Comp.	IC_50_ AChE [µM]	IC_50_ BChE [µM]	Ref.
**75**	24.25 *±* 2.97	0.002 *±* 0.0014	[91]
**94**	17.95 *±* 0.90	17.51 *±* 0.21	[92]
**95**	28.16 *±* 0.98	1.69 *±* 0.17	[92]
**96**	24.75 *±* 0.17	>500	[92]
**tacrine**	0.124 ± 0.02	7.8 ± 0.06	[91]
**rivastigmine**	56.10 *±* 1.41	38.40 *±* 1.97	[92]

**Table 3:** Biological activity of selected amidrazones studied in vivo.

Comp.	Activity	Animal Model	Dose	Effect	Reference Drug	Ref.
**23**	anti-inflammatory	xylene-induced ear edema test in mice	100 mg/kg	92.45% edema reduction	indomethacin 89.38% reduction, ibuprofen 87.36% reduction	[25]
**25**	anti-inflammatory	xylene-induced ear edema test in mice	50 mg/kg	93.56% edema reduction	indomethacin 45.23% reduction, ibuprofen 29.56% reduction	[26]
**26**			50 mg/kg	81.65% edema reduction		
**30**	antibacterial	MRSA-infected *C. elegans*	20 mg/mL	reduction in the MRSA burden by ~90%	vancomycin ~90% reduction	[30]
		MRSA murine skin infection	2% suspension	73% reduction in MRSA burden	fusidic acid 78% reduction	
		MRSA-infected mice	20 mg/kg	77% reduction in MRSA burden	vancomycin 66% reduction	
**31**		MRSA-infected *C. elegans*	20 mg/mL	reduction in the MRSA burden by ~90%	vancomycin ~90% reduction	
		MRSA murine skin infection	2% suspension	71% reduction in MRSA burden	fusidic acid 78% reduction	
**55**	anti-inflammatory	carrageenan-induced rat hind paw edema	21 mg/kg	65–73% edema reduction (0.5–2 h)	diclofenac 50–58% edema reduction (0.5–8 h)	[50]
			42 mg/kg	38–60% edema reduction (0.5–2 h)		
	antinociceptive	hot-plate test in mice	21 mg/kg	analgesic effect (0.5–2 h)	morphine analgesic effect(0.5–1 h)	
			42 mg/kg	analgesic effect (0.5–2 h)		
**60**	anti-inflammatory	carrageenan-induced rat hind paw edema	65 mg/kg	89.3% edema reduction	indomethacin 46% edema reduction	[55]
**61**			65 mg/kg	87.7% edema reduction		
**62**			61 mg/kg	80.7% edema reduction		
**63**			61 mg/kg	79.5% edema reduction		
**65**	anti-inflammatory neonatal sepsis treatment	LPS-induced sepsis in neonatal mice	50 mg/kg	reduction in anxiety-like behavior and cognitive disorders in adult life	-	[58]

**Table 4:** Mechanism of action and molecular targets of select amidrazone derivatives.

Comp.	Activity	Mechanism	Ref.
**AG**	anti-inflammatory	suppression of oxidative stress, inhibition of IL-1β, IL-6, and Foxp3 mRNA upregulation	[57]
**1**	antituberculosic	inhibiting protein synthesis via direct binding to the bacterial ribosomal subunit	[15]
**8**	antibacterial	inhA inhibition	[19]
**27**	antibacterial	inhibition of DHFR protein	[27]
**28**	antibacterial	interaction with *E. coli* FabH-CoA receptor.	[28]
**29**	antibacterial	interaction with β-ketoacyl-ACP synthase III (FabH)	[29]
**30**	antibacterial	inhibitor of undecaprenyl diphosphate phosphatase and undecaprenyl diphosphate	[30]
**38–39**	antifungal	interaction with DNA (intercalation)	[37]
**43**	antifungal	inhibition of 14-α-demethylase (CYP51)	[40]
**46**	antigiardial	inhibition of adherence of trophozoides	[42]
**47**	cytoprotective	inhibition of R15A, inhibition of dephosphorylation of enzyme eIF2α	[59]
**48**	cytoprotective	inhibition of R15B, inhibition of dephosphorylation of enzyme eIF2α	[66]
**48–50**	antiparasitic	binding trypanothione reductase enzyme	[45]
**53**	antiviral	inhibition of HIV-RT	[48]
**54**	anti-inflammatory	decreasing production of TNF-α	[49]
**55**	anti-inflammatory	decreasing production of IL-6	[50]
**56**	anti-inflammatory	decreasing production of TNF-α	[51]
**57**	anti-inflammatory	G1 phase arrest	[52]
**58**	anti-inflammatory	decreasing production of IL-6	[54]
**60–63**	anti-inflammatory	inhibition of COX-1 and COX-2	[55]
**64**	antarthritic	inhibition expression of ASIC1a protein	[56]
**65**	anti-inflammatory	inhibition of NFκB activation	[58]
**66**	cytoprotective	inhibition of R15A, inhibition of dephosphorylation of enzyme eIF2α	[59]
**72**	antitumor	tyrosine kinase brc-abl inhibitor	[71]
**73**	antitumor	tyrosine kinase brc-abl inhibitor	[72]
**74**	antitumor	tyrosine kinase brc-abl inhibitor	[73]
**75**	antitumor	tyrosine kinase brc-abl inhibitor	[74]
**76**	antitumor	tyrosine kinase brc-abl inhibitor	[75]
**79**	antitumor	phosphatidylinositol 3-kinase inhibitor	[77]
**81**	antitumor	inhibition of tubulin polymerization, colchicine binding	[78]
**82**	antitumor	inhibition of ribosomal kinase RSK2	[79]
**92**	enzyme inhibition	furin inhibitor, trypsin inhibitor	[89]
**93**	enzyme inhibition	furin inhibitor, thrombin inhibitor	[89]
**75**	enzyme inhibition	BChE inhibitor	[91]
**94**	enzyme inhibition	AChE and BChE inhibitor	[92]
**95–96**	enzyme inhibition	BChE inhibitor	[92]

**Table 5:** The toxicity of selected amidrazones in various animal or normal cell models.

**Comp.**	**Animal Model**	**Time**	**Toxicity**	**Ref.**
**18**	brine shrimp	24 h	IC_50_ > 50 µg/mL	[23]
**19**	brine shrimp	24 h	IC_50_ > 50 µg/mL	[23]
**20**	brine shrimp	24 h	IC_50_ > 12.5 µg/mL	[23]
**21**	brine shrimp	24 h	IC_50_ > 12.5 µg/mL	[23]
**43**	zebrafish embryos	96 h	LC_50_ = 8.2 µg/mL	[40]
**55**	Swiss mice	-	LD_50_ = 417 mg/kg	[50]
**78**	brine shrimp	24 h	IC_50_ > 50 µg/mL	[23]
**Comp.**	**Studied cells**	**Origin**	**Toxicity**	**Ref.**
**2**	Vero	monkey	IC_50_ = 28.7 µM	[17]
**3**	Vero	monkey	IC_50_ = 23.1 µM	[17]
**4**	Vero	monkey	IC_50_ = 27.8 µM	[17]
**5**	Vero	monkey	IC_50_ = 298 µM	[17]
**6**	fibroblasts	human	IC_50_ = 10.39 µg/mL	[18]
**7**	fibroblasts	human	IC_50_ = 3.29 µg/mL	[18]
**28**	HEK 293T	human	IC_50_ = 56.39 µmol/L	[28]
**32**	fibroblasts	mice	IC_50_ = 41.8 µg/mL	[31]
**43**	MRC-5	human	IC_50_ = 2.5 µg/mL	[40]
**23**	LO2	human	IC_50_ = 18.1 µg/mL	[25]
**30–31**	HRT-18	human	IC_50_ > 32 µg/mL	[30]
**44**	macrophages	mice	IC_50_ = 79.59 µM	[41]
**45**	macrophages	mice	IC_50_ = 423.33 µM	[41]
**46**	RAW264.7	mice	IC_50_ = 17.1 µM	[42]
**48–50**	J774.A1	mice	IC_50_ > 10 μM	[45]
**51**	PBMC	human	IC_50_ > 100 µg/mL	[46]
**54**	PBMC	human	IC_50_ > 100 µg/mL	[49]
**56**	PBMC	human	IC_50_ > 10 µg/mL	[51]
**57**	PBMC	human	IC_50_ > 50 µg/mL	[52]
**58–59**	PBMC	human	IC_50_ > 100 µg/mL	[54]
**64**	chondrocytes	rat	IC_50_ > 25 µM	[56]
**72**	fibroblasts	human	IC_50_ > 50 µM	[71]
**76**	fibroblasts	human	IC_50_ = 15 µM	[75]
**83**	Vero	monkey	IC_50_ > 100 µM	[80]
**87**	Vero	monkey	IC_50_ > 611.09 µM	[84]
**88**	ARPE-19	human	IC_50_ = 38.82 µM	[85]
**89**	ARPE-19	human	IC_50_ = 41.23 µM	[85]
**90**	MRC-5	human	IC_50_ = 58.9 µM	[86]
**91**	PBMC	human	IC_50_ > 25 µg/mL	[49]

ARPE-19—human epithelial cell line derived from retina; HEK 293T—human embryonic kidney cells; HRT—human colorectal cells; J774.A1—mice macrophages; LO2—human hepatocytes; MRC5—human lung fibroblasts; PBMC—human peripheral mononuclear cells; RAW264.7—mice macrophages; Vero—monkey kidney epithelial cells.

## Data Availability

Not applicable.

## Abbreviations

AChE	acetylcholinesterase
BChE	butyrylocholinesterase
CNS	central nervous system
COX	cyclooxygenase
EC_50_	half maximal effective concentration
HIV-RT	HIV reverse transcriptase
IC_50_	half-maximal inhibitory concentration
IL-6	interleukin-6
LD_50_	dose which causes the death of 50% of a group of test animals
LPS	lipopolysaccharide
MBC	minimal bactericidal concentration
MIC	minimal inhibitory concentration
MRSA	methicillin-resistant *Staphylococcus aureus*
MSSA	methicillin-susceptible *Staphylococcus aureus*
PBMC	peripheral blood mononuclear cell
TNF-α	tumor necrosis factor
